# Enhanced Low-Temperature Corn Straw Degradation Using a Synthetic Microbial Mixture

**DOI:** 10.3390/life16030402

**Published:** 2026-03-02

**Authors:** Yi Fang, Jiaqi Li, Susu Yu, Xuhong Ye, Li Zhang, Hongtao Zou

**Affiliations:** 1 College of Land and Environment, Shenyang Agricultural University, Dongling Road No. 120, Shenyang 110866, China; 13658424354@163.com (Y.F.); lijiaqi2023@syau.edu.cn (J.L.); 15940274591@163.com (S.Y.); 15390649309@163.com (L.Z.); 2 Northeast Key Laboratory of Conservation and Improvement of Cultivated Land (Shenyang), Ministry of Agriculture and Rural Affairs, Shenyang 110866, China; 3 National Engineering Research Center for Efficient Utilization of Soil and Fertilizer Resources, Shenyang 110866, China

**Keywords:** low-temperature degradation, corn straw, synthetic microbial mixture, cellulase activity

## Abstract

The structural stability of lignocellulosic fibers in crop straw presents a significant challenge to its short-term biodegradation in natural environments, particularly in the cold regions of northern China. To isolate low-temperature straw-degrading bacteria, we selectively enriched microorganisms from straw-amended soils using lignocellulose as the sole carbon source. Three strains were isolated and identified: *Stenotrophomonas* sp. X24, *Flavobacterium* sp. X26, and *Erwiniaceae bacterium* X27. These strains were capable of growth and maize straw degradation within a 4–20 °C range and exhibited key cellulolytic activities (CMCase, FPase, and β-glucosidase). A synthetic three-strain mixture was assembled by combining these isolates in equal proportions. Solid-state fermentation (12 °C, 45 days) was used to assess straw degradation efficacy, while separate enzyme production experiments (12 °C, 3 days) were conducted to evaluate key cellulolytic activities and subsequently optimize culture conditions. The three-strain mixture achieved a net straw degradation rate of 30.93 ± 1.05%. Furthermore, optimization of culture conditions enhanced the carboxymethyl cellulase activity (CMCase) to a maximum of 24.51 ± 0.97 U/mL. The study demonstrates that the three-strain synthetic microbial mixture effectively degrades straw at low temperatures, offering a promising microbial resource to improve straw utilization and soil fertility in cold regions.

## 1. Introduction

Corn stover, a major agricultural byproduct, contributes to carbon sequestration [1], improves soil properties, and supports soil ecology [2]. However, this residue primarily consists of plant cell walls composed of cellulose, hemicellulose, and lignin, which are highly recalcitrant to degradation [3]. This poses significant challenges, particularly in Northeast China, where the average winter temperature is −11.2 °C (China Meteorological Administration, 2023), which substantially inhibits microbial metabolic activity.

Traditional treatment methods for agricultural waste disposal, such as open burning, and those for industrial cellulose recovery, such as chemical pretreatments using strong acids or alkalis, remain widely employed [4]. However, these approaches lead to severe environmental consequences, including air pollution, water contamination, and ecosystem hazards [5]. In contrast, biological strategies, which utilize microorganisms with straw-degrading capabilities, can effectively address the limitations of traditional treatment methods and mitigate environmental pollution [6]. The microbial degradation of straw relies on the secretion of various hydrolytic enzymes, particularly cellulases such as exoglucanases, endoglucanases, and β-glucosidases [7]. Filter paper and sodium carboxymethyl cellulose (CMC) are commonly used as standard, chemically defined substrates representing crystalline and amorphous cellulose, respectively [8,9]. These substrates allow for the specific enrichment and isolation of bacteria with high exoglucanase and endoglucanase activities. Exoglucanases act processively on the cellulose chain ends, releasing cellobiose as the main product [10]. Endoglucanases target amorphous regions, hydrolyzing internal β-1,4-glycosidic bonds to release oligosaccharides and create new chain ends. Ultimately, β-glucosidases convert cellobiose and oligosaccharides into glucose, thereby completing the entire degradation process [2]. The synergistic action of these enzymes enables microbes to efficiently break down recalcitrant cellulose into glucose, thereby facilitating straw resource utilization [11]. However, microbial metabolism is influenced by various factors, with temperature being a primary determinant of degradation activity [12]. In Northeast China, cold stress significantly hinders the natural biodegradation of abundant agricultural straw residues. Undegraded straw reduces crop growth in the following season and fosters pests and diseases [13]. Therefore, it is of great importance to identify microorganisms with cellulose-degradation capability at low temperatures to accelerate straw decomposition and enhance soil fertility in colder regions.

Microorganisms investigated in previous studies primarily include fungi and bacteria. Among these, fungi produce substantial amounts of cellulolytic enzymes, playing a predominant role in the straw degradation process [14]. However, individual strains often possess limited environmental adaptability and incomplete enzyme systems. Consequently, compared to single strains, composite microbial agents demonstrate greater adaptability and exhibit synergistic effects, leading to superior degradation efficacy [15]. Within such agents, diverse microorganisms interact with the degradation environment by secreting specific enzymes. This interaction facilitates the formation of a specialized microbial consortium, which collectively utilizes its distinct functions to achieve efficient straw degradation [16]. The conventional method for constructing microbial inoculants involves the initial screening of individual high-efficiency strains using pure culture techniques. Subsequently, composite inoculants are formulated for enhanced degradation efficiency. This process typically focuses on the optimization of combination strategies and the utilization of synergistic effects among diverse microorganisms [17]. For example, a composite microbial agent (CB) was formulated using both *Phanerochaete* and *Chrysosporium*. After a 20-day degradation period at an optimal temperature of 32 °C, the degradation ratios of lignin, cellulose, and hemicellulose in corn straw were 44%, 31%, and 48%, respectively, indicating the efficacy of synergistic microbial degradation [18]. While fungal systems have shown great efficacy in degrading straw, bacterial consortia often exhibit broader environmental adaptability and richer metabolic diversity [19].

Therefore, this study aimed to assemble a synthetic microbial mixture by leveraging cooperative interactions among different microbial strains. Straw-returned soil served as the source material for isolation. Three cold-adapted bacterial strains with high-efficiency cellulolytic capabilities were isolated and identified: *Stenotrophomonas* sp. X24, *Flavobacterium* sp. X26, and *Erwiniaceae bacterium* X27. Subsequently, these strains were assembled into a synthetic microbial mixture. In this study, we evaluated the efficacy of this three-strain mixture in degrading lignocellulosic biomass, potentially through synergistic enzymatic mechanisms. By applying this synthetic microbial mixture during winter straw incorporation, we aimed to accelerate low-temperature straw decomposition and enhance soil fertility in cold regions.

## 2. Materials and Methods

### 2.1. Isolation and Screening of Low-Temperature Corn Straw-Degrading Bacteria

The screening of bacteria for corn straw degradation was conducted in three stages: sampling and cultivation, primary screening, and secondary screening. Soil samples were collected from surface layers rich in humus at the Long-term Corn Straw Return Experimental Station of Shenyang Agricultural University in Shenyang, Liaoning Province (41.82° N, 123.56° E, elevation 43 m). Samples were preserved at 4 °C for subsequent analysis. Detailed information on the physicochemical properties of the soils is provided in Table S1.

#### 2.1.1. Preliminary Screening of Cellulose-Degrading Bacteria

Ten grams of soil and 90 mL of sterile distilled water were added to a 250 mL Erlenmeyer flask. The flask was agitated at 180 rpm for 30 min, followed by a 30-min static incubation period. Then, 1 mL of the supernatant was inoculated into 100 mL of Hutchinson’s liquid medium (KH_2_PO_4_ 0.1% (*w*/*v*), FeCl_3_ 0.001% (*w*/*v*), MgSO_4_·7H_2_O 0.03% (*w*/*v*), NaNO_3_ 0.25% (*w*/*v*), CaCl_2_ 0.01% (*w*/*v*), NaCl 0.01% (*w*/*v*)), containing a Whatman No. 1 filter paper strip [20]. Due to its high crystallinity [8], this filter paper was used as the sole carbon source for the targeted enrichment of cellulolytic microorganisms. The incubation was performed at 20 °C to serve as a selective threshold for cold-tolerant bacteria. To ensure stable enrichment, 10% (*v*/*v*) of the culture was serially transferred into fresh medium upon visible disintegration of the filter paper for a total of three consecutive passages. Subsequently, the culture derived from the decomposed filter paper was transferred to a CMC liquid medium (1.5% (*w*/*v*) sodium carboxymethyl cellulose (CMC-Na; Sinopharm Chemical Reagent Co., Ltd., Shanghai, China), NH_4_NO_3_ 0.1% (*w*/*v*), yeast extract 0.1% (*w*/*v*), MgSO_4_·7H_2_O 0.05% (*w*/*v*), and KH_2_PO_4_ 0.1% (*w*/*v*)) for further cultivation. The culture medium was incubated with agitation at 20 °C and 180 rpm for three days to facilitate the proliferation of cellulolytic bacteria enriched in the preliminary screening. Under sterile conditions, the bacterial suspension was subsequently serially diluted from 10^−4^ to 10^−6^. The diluted samples were then plated on CMC solid medium supplemented with 2% agar. Each dilution series was performed in triplicate. Morphologically distinct bacterial colonies were isolated for subculturing, ultimately leading to the purification of single pure cultures. These purified isolates were stored at 4 °C and designated for subsequent secondary screening to evaluate their cellulolytic efficiency.

#### 2.1.2. Secondary Screening of Cellulose-Degrading Bacteria

A total of 50 single colonies with distinct morphologies on the agar plates were selected. The qualitative CMC hydrolyzing potential was screened using a Congo red staining assay. Based on their halo-to-colony diameter (D/d) ratios, the strains were ranked in descending order, and the top ten were selected. To evaluate low-temperature adaptability, these strains were inoculated onto CMC agar and incubated at 4, 8, 12, 16, and 20 °C. The observation periods were set according to the temperatures: 7 days at 20 °C, 14 days at 12–16 °C, and 30 days at 4–8 °C. Growth capability was determined by the presence of visible colonies as observed by the naked eye (Table S2). Only the strains showing visible growth across the entire 4–20 °C range were selected and subjected to further physiological and biochemical characterization.

### 2.2. Identification of Isolated Strains

#### 2.2.1. Morphological, Physiological, and Biochemical Characterization

The selected bacterial strains were inoculated onto Luria-Bertani (LB) agar and incubated at 20 °C for 48 h to obtain fresh biomass. Subsequently, the strains were characterized through physiological and biochemical assays following the protocols outlined in *Bergey’s Manual of Systematic Bacteriology* to determine positive or negative results. All assays were performed in biological triplicates to ensure reproducibility. A total of thirteen microbiological and biochemical assays were conducted, including Gram staining (crystal violet-iodine staining) [21]; VP test (Barritt’s reagent) [22]; Oxidation-Fermentation (O-F) test (Hugh-Leifson’s two-tube method) [23]; indole production (Kovacs’ reagent) [24]; gelatin liquefaction (stab culture in gelatin medium) [25]; catalase activity (hydrogen peroxide) [26]; oxidase activity (dimethyl-*p*-phenylenediamine dihydrochloride) [27]; nitrate reduction (*p*-aminobenzenesulfonic acid and α-naphthylamine) [28]; citrate utilization (Simmons’ citrate agar slant) [29]; methyl red test (methyl red indicator) [30]; amylase activity (starch hydrolysis on iodine-stained agar plates) [31]; motility (semi-solid agar stab inoculation) [32]; and lipase activity (Tween 80 hydrolysis precipitate formation) [33].

#### 2.2.2. Molecular Identification of Strains

To confirm the identity of the isolated strains, molecular analysis based on 16S rRNA gene sequencing was performed. Total DNA was extracted from the bacterial strains using the Tiangen bacterial genomic DNA extraction kit (Tiangen Biotech Co., Ltd., Beijing, China). Approximately 50 ng of extracted DNA was used as a template for PCR amplification. The 16S rRNA gene was amplified by PCR using the universal primers 27F and 1492R [34]. The amplification program consisted of an initial denaturation at 94 °C for 5 min; followed by 30 cycles of 94 °C for 30 s, 52 °C for 30 s, and 72 °C for 90 s; and a final extension at 72 °C for 10 min. The purified amplicons were sequenced bidirectionally at a commercial facility (Sangon Biotech, Shanghai, China). Sequence quality control was performed by visual inspection of chromatograms and trimming of low-quality ends using BioEdit (v7.0.9). Forward and reverse reads were subsequently assembled to generate a consensus sequence. The resulting consensus sequence for each strain was used for taxonomic identification. For phylogenetic analysis, 16S rRNA gene sequences of closely related type strains were retrieved from the LPSN (https://lpsn.dsmz.de/; accessed on 22 February 2026) and NCBI GenBank database (https://www.ncbi.nlm.nih.gov/; accessed on 22 February 2026). Sequences were aligned using the MUSCLE program as implemented in MEGA (version 11). Alignment quality was verified by visual inspection, and terminal gaps were truncated to ensure that only homologous positions were compared in the final phylogenetic analysis. A Neighbor-Joining (NJ) tree was constructed to determine the taxonomic positions of the isolates using the Kimura 2-parameter (K2P) substitution model. The robustness of the tree topology was evaluated using bootstrap analysis with 1000 replicates. Positions containing less than 95% site coverage were eliminated, resulting in a final dataset of 1387 positions.

### 2.3. Assembly of the Synthetic Cellulolytic Microbial Mixture

Based on the screening results (Table S2), the three cold-adapted strains (X24, X26, and X27) were selected for the subsequent assembly of the synthetic microbial mixtures. Preliminary experiments were conducted to determine the growth characteristics of each bacterial strain. The three strains were individually inoculated into three separate Erlenmeyer flasks containing fresh CMC liquid medium in triplicate. The strains were incubated at 30 °C and 180 rpm for optimal biomass accumulation, and their growth curves (Figure S1) were established by measuring A_600_ at two-hour intervals. Based on these profiles, inoculation times were staggered to synchronize the harvest of all strains during their exponential phase [35]: 24 h for strain X24, 16 h for strain X26, and 8 h for strain X27, respectively. Following incubation, a 5 mL aliquot of each bacterial culture was collected and centrifuged at 8000 rpm (6000× *g*) for 5 min, after which the supernatant was discarded to remove extracellular metabolites and potential cell lysis products. The resulting cell pellets were washed twice with sterile physiological saline (0.85% *w*/*v* NaCl) and resuspended in the same solution. Finally, each cell suspension was adjusted to a standardized optical density (A_600_) of approximately 1.0 to serve as working inocula. The standardized bacterial suspensions were combined in equal volumes based on a full factorial design to assemble four distinct synthetic microbial mixtures: Mixture A (X24 and X26, 1:1 *v*/*v*), Mixture B (X24 and X27, 1:1 *v*/*v*), Mixture C (X24, X26, and X27, 1:1:1 *v*/*v*/*v*), and Mixture D (X26 and X27, 1:1 *v*/*v*). These four mixtures, representing all possible two-strain and three-strain combinations, were subsequently subjected to enzyme activity assays to evaluate their synergistic cellulolytic potential.

### 2.4. Measurement of Cellulolytic Activities

The individual strains (X24, X26, and X27) and the four synthetic microbial mixtures were inoculated in triplicate at 10% (*v*/*v*) into a specific liquid enzyme production medium (KH_2_PO_4_ 0.2% (*w*/*v*), (NH_4_)_2_SO_4_ 0.14% (*w*/*v*), MgSO_4_·7H_2_O 0.03% (*w*/*v*), CaCl_2_ 0.03% (*w*/*v*), FeSO_4_·7H_2_O 0.0005% (*w*/*v*), CMC-Na 0.5% (*w*/*v*), peptone 0.5% (*w*/*v*), beef extract 0.25% (*w*/*v*), pH 7.0). This medium was formulated to support initial biomass accumulation via rich nitrogen sources, thereby extending the exponential and stationary growth phases [36]. In accordance with the temperature coefficient (*Q*_10_) principle, metabolic rates slow down at low temperatures [37]; therefore, the incubation period was extended to 72 h at 12 °C. Following incubation, the fermentation broth was centrifuged at 5000 rpm for 10 min at 4 °C. The resulting supernatant was collected as the crude enzyme extract. The total protein concentration (mg/mL) of the crude enzyme extract was determined using the Bradford method [38], with bovine serum albumin (BSA) as the standard. Subsequently, the activities of the following three enzymes were assayed using the modified 3,5-dinitrosalicylic acid (DNS) colorimetric method [39].

For filter paper hydrolyzing activity (FPase), the reaction mixture contained 1.5 mL of 50 mM citrate buffer (pH 4.8), one strip of Whatman No. 1 filter paper (1.0 × 6.0 cm, approx. 50 mg), and 0.5 mL of crude enzyme extract. The reaction was performed at 50 °C for 60 min following standard IUPAC guidelines. For endoglucanase activity (CMCase), the reaction mixture consisted of 1.5 mL of 1% (*w*/*v*) CMC-Na (Sinopharm) dissolved in the same buffer and 0.5 mL of crude enzyme extract. The reaction was incubated at 50 °C for 30 min. For β-glucosidase activity, the assay was conducted following the same procedure as CMCase, except that the 1% (*w*/*v*) CMC-Na solution was replaced with a 1% (*w*/*v*) salicin (Sinopharm) solution. All reactions were terminated by adding 1.5 mL of DNS reagent, followed by incubation in a boiling water bath for 10 min. After cooling to room temperature, the reaction mixtures were diluted to a final volume of 25 mL with distilled water. The absorbance was measured at 540 nm (A_540_) using an N2S Visible Spectrophotometer (Shanghai Yidian Analytical Instrument Co., Ltd., Shanghai, China). An enzyme blank (extract added after DNS) and a substrate-only negative control were included to subtract background reducing sugars. All assays were performed in three independent biological replicates.

One unit (U) of enzyme activity was defined as the amount of enzyme that catalyzes the production of 1 µmol of glucose equivalents per minute under the specified assay conditions. The specific activity (U/mg) was calculated as the units of activity (U) per milligram of total protein in the crude enzyme extract. The glucose standard curve is shown in Figure S2. The specific activity (U/mg) was calculated using the following formula:

$$Specific\;Activity(U/mg)=\frac{C\times V}{T\;\times M_{protein}}$$

where *C* is the net concentration of glucose equivalents (µmol/mL) produced (calculated from the sample absorbance minus the absorbances of both controls), as determined by the DNS assay against a glucose standard curve; *V* is the final diluted volume of the reaction mixture (mL); *T* is the reaction time (minutes); and *M_protein_* is the mass of total protein added to the reaction mixture (mg).

### 2.5. Determination of Corn Straw Degradation Efficiency

The corn straw degradation test was performed at 12 °C for 45 days to determine the degradation efficiency under low-temperature conditions. The corn straw was cut into 2–3 cm segments and oven-dried at 80 °C to constant weight. For liquid fermentation, the straw was ground to pass through a 40-mesh sieve, while 2–3 cm segments were used for solid-state fermentation. The degradation process was monitored by calculating the weight loss of the corn straw. All treatments, including uninoculated controls, were performed in three biological replicates. Detailed experimental parameters for comparing the two fermentation modes are summarized in Table S7.

#### 2.5.1. Liquid Fermentation of Straw-Degrading Bacteria

Two grams of sterilized, processed corn straw (*M*_0_, dry weight equivalent) and five milliliters of the standardized bacterial suspension were inoculated into 100 mL of liquid fermentation medium (peptone 0.2% (*w*/*v*), MgSO_4_·7H_2_O 0.05% (*w*/*v*), KH_2_PO_4_ 0.1% (*w*/*v*), NaCl 0.05% (*w*/*v*), CaCl_2_ 0.02% (*w*/*v*), and yeast extract 0.05% (*w*/*v*), pH 7.0). The cultures were incubated at 12 °C with agitation at 120 rpm for 45 days. Following incubation, the cultures were centrifuged at 6000 rpm (3375× *g*) for 10 min to pellet the insoluble residues. The straw residues were washed twice with a 0.1 M HCl solution and then rinsed with distilled water. The washed residues were then dried to a constant weight at 105 °C, and the residual straw mass was weighed (*M*_1_). The degradation efficiency was expressed as the net weight loss percentage, calculated as follows:

$$Net\;degradation(\%)=\frac{M_{ck}\;-\;M_{1}}{M_{0}}\times 100$$

where *M_ck_* is the residual mass of the straw in the blank control group.

#### 2.5.2. Solid-State Fermentation of Straw-Degrading Bacteria

Solid-state fermentation was carried out in 250 mL conical flasks sealed with breathable silicone plugs. Ten grams of sterilized corn straw segments (*M*_0_, dry weight equivalent) were mixed with 20 mL of solid-state fermentation nutrient medium (peptone 1% (*w*/*v*), MgSO_4_·7H_2_O 0.25% (*w*/*v*), KH_2_PO_4_ 0.5% (*w*/*v*), NaCl 0.25% (*w*/*v*), CaCl_2_ 0.1% (*w*/*v*), and yeast extract 0.25% (*w*/*v*), pH 7.0) and inoculated with 2 mL of bacterial suspension. To maintain humidity, the flasks were placed in a sealed container containing saturated KNO_3_ solution and incubated at 12 °C for 45 days. During fermentation, the substrate was stirred every 5 days. Simultaneously, substrate moisture was kept above 50% by gravimetrically replenishing evaporative water loss with sterile deionized water. Finally, the residues were washed with 0.1 M HCl and distilled water, and dried to constant weight (*M*_1_) to assess the degradation efficiency of the straw during the process.

### 2.6. Optimization of Culture Conditions for Cellulase Production

Based on cellulolytic activity results (Section 3.3) and the degradation efficiency (Section 3.4), the three-strain mixture exhibited significantly higher performance compared to the individual strains and other microbial mixtures. Consequently, it was selected for optimization to determine the optimal culture conditions for enzyme production. Single-factor experiments were conducted using the liquid enzyme production medium described in Section 2.4. The inoculum used for this experiment was the three-strain mixture, prepared by combining equal volumes of the standardized bacterial suspensions described in Section 2.3. The experiment investigated four independent variables, each at five levels: inoculum size (2%, 4%, 6%, 8% and 10% *v*/*v*); incubation temperature (4, 8, 12, 16 and 20 °C); fermentation time (24, 48, 72, 96 and 120 h); and initial pH (5.0, 6.0, 7.0, 8.0 and 9.0). For each variable tested, all other parameters were maintained at their basal levels (Inoculum size 10% (*v*/*v*), Temperature 12 °C, Time 72 h, and pH 7.0). For pH optimization, the medium was adjusted to the respective values prior to sterilization. All fermentation experiments, including an uninoculated control, were performed in independent triplicates in an orbital shaker at 180 rpm. Following incubation, the CMCase activity was determined as described in Section 2.4 to identify the optimal conditions. Finally, the reported enzymatic activities were corrected by subtracting the background values measured in the uninoculated controls.

### 2.7. Statistical Analysis

All experiments were conducted in three independent biological replicates, and data are expressed as the mean ± standard deviation (SD). Statistical analyses were performed using R software (version 4.5.1). The normality of the data was assessed using the Shapiro-Wilk test, and the homogeneity of variances was verified using Levene’s test (via the ‘car’ package). Differences among groups were analyzed using one-way analysis of variance (ANOVA). Post-hoc multiple comparisons were conducted using Tukey’s honestly significant difference (HSD) test to determine significant differences between specific means. Differences were considered statistically significant at *p* < 0.05. Data processing was carried out with Microsoft Excel 2021, and graphical representations were generated using GraphPad Prism version 9.5, where error bars explicitly represent the standard deviation (SD).

## 3. Results

### 3.1. Screening of Cold-Adapted Cellulolytic Bacterial Strains

After 2–3 serial transfers, the filter paper gradually disintegrated, and microbial growth was observed at the interface between the filter paper strips and the culture medium. Cellulolytic bacteria were isolated on CMC solid medium using serial dilution plating. Based on colony morphology, including color, size, and shape, 43 isolates were selected and purified. Based on Congo red staining assays for cellulase activity, 10 strains were further screened and assigned identification numbers. Subsequently, cultivation at a temperature gradient of 4 °C, 8 °C, 12 °C, 16 °C, and 20 °C was performed to evaluate low-temperature adaptability. The results (Table S2) indicated distinct differences in the cold tolerance for growth among the strains. Notably, only strains X24, X26, and X27 demonstrated the ability to form visible colonies at the lowest tested temperature of 4 °C. In contrast, other strains exhibited limited cold tolerance. For instance, strains X4 and X23 showed no growth at 4 °C, while strains X14 and X17 failed to form colonies below 20 °C. Based on their capacity to grow at 4 °C, strains X24, X26, and X27 were selected to assemble the low-temperature synthetic microbial mixture.

### 3.2. Taxonomic Identification of the Selected Bacterial Strains

Based on morphological, physiological and biochemical characteristics (Table 1), strains X24, X26 and X27 were preliminarily identified as belonging to the genera *Stenotrophomonas*, *Flavobacterium* and *Pantoea*, respectively. To confirm this taxonomic assignment, the 16S rRNA genes of these strains were sequenced, yielding lengths of 1450 bp, 1417 bp, and 1443 bp, respectively. Their sequences were then compared with those of type strains in the NCBI database using BLAST. The results showed that strain X24 exhibited the highest sequence similarity (99.65%) to the type strain of *Stenotrophomonas maltophilia* (GenBank accession no. CP001111.1) (Table S3). Strain X26 exhibited the highest sequence similarity (99.01%) to the type strain of *Flavobacterium johnsoniae* (GenBank accession no. AB681010.1) (Table S4). Therefore, strain X24 was tentatively identified as *Stenotrophomonas* sp. and strain X26 as *Flavobacterium* sp. However, the identification of strain X27 was inconclusive. Although its closest match was *Pantoea rodasii* (GenBank accession no. MT733958.1) with 96% similarity, this value is below the 98.7% threshold recommended for species delineation. Consequently, phylogenetic trees were constructed for all three strains to further confirm their taxonomic status.

**Figure 1:**
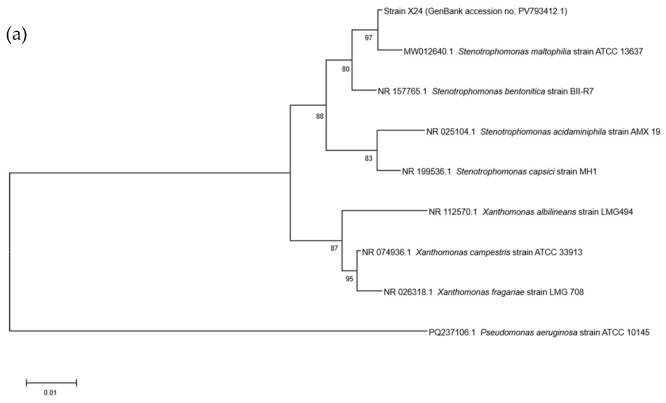

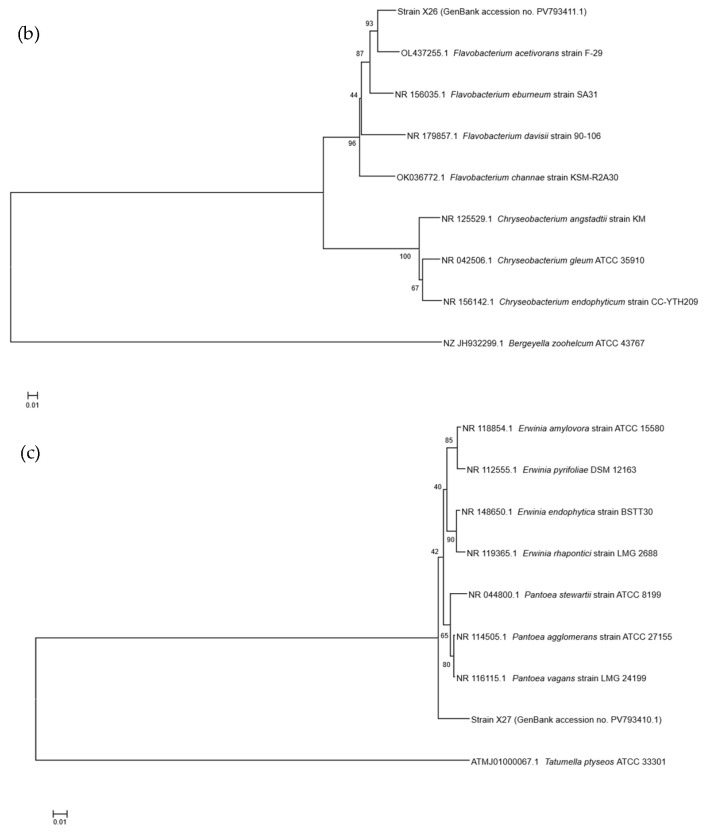
Phylogenetic trees showing the evolutionary placement of the screened strains based on 16S rRNA gene sequences. The trees were constructed using the Neighbor-Joining method. Numbers at the nodes indicate bootstrap percentages (from 1000 replicates). The scale bar represents nucleotide substitutions per site. (**a**) Strain X24 (*Stenotrophomonas* sp.); (**b**) Strain X26 (*Flavobacterium* sp.); (**c**) Strain X27 (*Erwiniaceae bacterium*).

The phylogenetic results (Figure 1) confirmed the preliminary identifications of strains X24 and X26, as they formed distinct clades with type strains of *Stenotrophomonas* and *Flavobacterium*, respectively. In contrast, strain X27 does not cluster unambiguously within either the *Pantoea* or the *Erwinia* clades. It forms a distinct lineage that is basal to both genera. Consequently, strains X24 and X26 were identified as *Stenotrophomonas* sp. (GenBank accession no. PV793412.1) and *Flavobacterium* sp. (GenBank accession no. PV793411.1). While strain X27 was provisionally classified as a member of the family *Erwiniaceae* (GenBank accession no. PV793410.1), it potentially represents a novel genus.

### 3.3. Cellulolytic Potential of Individual Strains and Synthetic Mixtures

**Figure 2:**
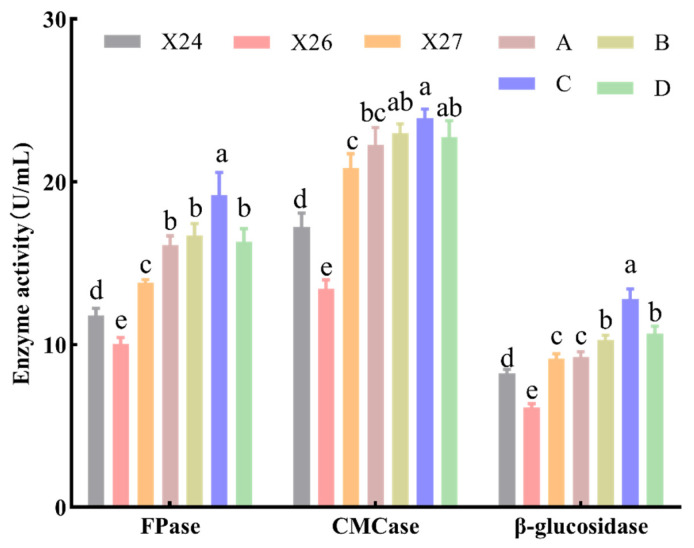
CMCase, FPase, and β-glucosidase activity of individual strains and synthetic microbial mixtures. Composition of synthetic microbial mixtures: A (X24 + X26), B (X24 + X27), C (X24 + X26 + X27), and D (X26 + X27). Strain identities: X24 (*Stenotrophomonas* sp.), X26 (*Flavobacterium* sp.), and X27 (*Erwiniaceae bacterium*). Data are shown as mean ± standard deviation (SD) (*n* = 3). Different lowercase letters indicate significant differences (*p* < 0.05, ANOVA with Tukey’s test).

To evaluate the hydrolytic potential of the individual strains and the synthetic microbial mixtures, the cellulolytic activities (CMCase, FPase, and β-glucosidase) were determined in the specific enzyme production medium. The results (Figure 2) showed that the cellulolytic activities differed significantly among the strains (*p* < 0.05). Among them, strain X27 exhibited significantly higher activities for all tested enzymes compared to the other strains, with a CMCase of 20.85 ± 0.71 U/mL, FPase of 13.81 ± 0.16 U/mL, and β-glucosidase activity of 9.14 ± 0.24 U/mL. While strain X27 demonstrated the highest individual activity, all four synthetic microbial mixtures exhibited significantly higher cellulolytic activities compared to the individual strains (*p* < 0.05). Notably, the cellulolytic activities of the three-strain mixture (Mixture C) were significantly superior to those of the other mixtures. Specifically, Mixture C achieved a CMCase of 23.91 ± 0.47 U/mL, an FPase of 19.17 ± 1.15 U/mL, and a β-glucosidase activity of 12.80 ± 0.51 U/mL. This provided a strong scientific basis for selecting the three-strain mixture as the subject for subsequent optimization (Section 2.6).

### 3.4. Degradation Efficiency in Solid and Liquid Fermentation

**Figure 3:**
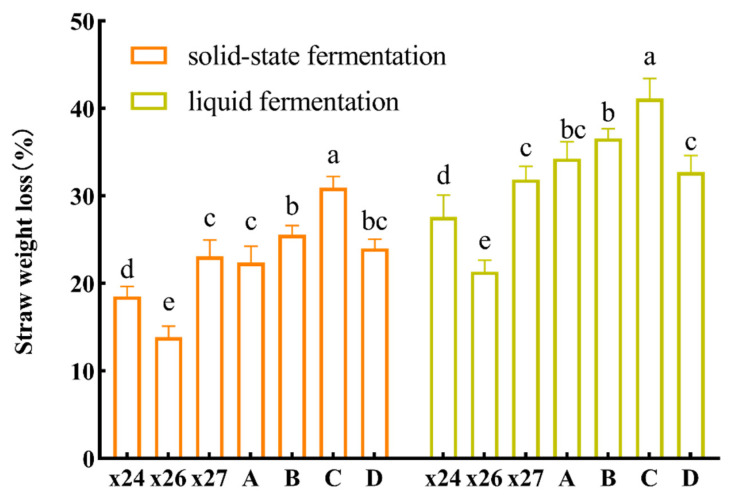
Degradation efficiency of corn straw during solid-state and liquid fermentation by individual strains and synthetic microbial mixtures. Composition of synthetic microbial mixtures: A (X24 + X26), B (X24 + X27), C (X24 + X26 + X27), and D (X26 + X27). Strain identities: X24 (*Stenotrophomonas* sp.), X26 (*Flavobacterium* sp.), X27 (*Erwiniaceae bacterium*). Data are shown as mean ± standard deviation (SD) (*n* = 3). Different lowercase letters indicate significant differences (*p* < 0.05, ANOVA with Tukey’s test).

The results showed that all seven experimental groups exhibited varying degrees of corn straw degradation (Figure 3). Consistent with the results of the cellulolytic activity assays, strain X27 achieved the highest degradation efficiency among the individual strains in both solid-state (23.08 ± 1.15%) and liquid-state (31.88 ± 1.22%) fermentation, significantly surpassing the others (*p* < 0.05). Additionally, the degradation efficiencies of all microbial mixtures were significantly higher than those of the individual strains. Among them, the three-strain mixture (Mixture C) achieved the highest values, reaching 30.93 ± 1.05% in solid-state and 41.11 ± 1.90% in liquid-state fermentation.

### 3.5. Optimization of Enzymatic Production Conditions for the Three-Strain Mixture

**Figure 4:**
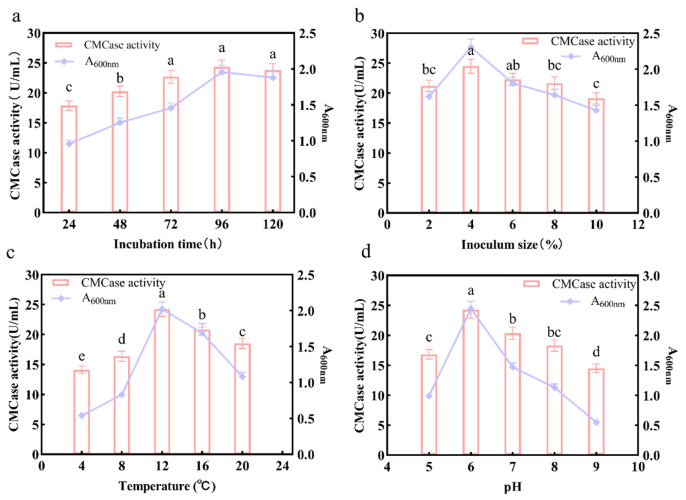
Effects of cultivation parameters on CMCase activity of the three-strain mixture (strains X24, X26 and X27) in single-factor experiments. Strain identities: X24 (*Stenotrophomonas* sp.), X26 (*Flavobacterium* sp.), X27 (*Erwiniaceae bacterium*). (**a**) Incubation time; (**b**) Inoculum size; (**c**) Temperature; (**d**) pH. Data are shown as mean ± standard deviation (SD) (*n* = 3). Different lowercase letters indicate significant differences (*p* < 0.05, ANOVA with Tukey’s test).

Single-factor optimization of culture conditions (incubation time, inoculum size, temperature, and pH) was performed to determine optimal enzyme production parameters for the three-strain mixture. Biomass accumulation (A_600_) and CMCase activity both reached a plateau after 72 h of cultivation (Figure 4a). Although the maximal CMCase activity (24.35 ± 0.91 U/mL) was observed at 96 h, no significant difference was found compared to 72 h (*p* > 0.05). Regarding inoculum size, maximum enzymatic activity (24.51 ± 0.97 U/mL) was achieved at a reduced level of 4% (Figure 4b). The optimal temperature for CMCase production was 12 °C, coinciding with peak values for both enzyme activity (24.22 ± 0.97 U/mL) and biomass accumulation. Significantly reduced activity (*p* < 0.05) occurred at elevated temperatures (Figure 4c). Neutral to slightly acidic conditions enhanced cellulolytic activity, yielding maximal CMCase (24.27 ± 1.17 U/mL) at pH 6.0 (Figure 4d). Based on these results, the optimized culture conditions were established as: incubation time of 72 h, inoculum size of 4%, temperature of 12 °C, and pH 6.0.

## 4. Discussion

Achieving efficient and environmentally friendly degradation of lignocellulosic biomass at low temperature remains a significant challenge in sustainable development. The key to this process lies in disrupting the complex structural barriers of lignocellulose [40]. Soil, as a vital reservoir of microbial diversity [41], hosts microbial communities that have evolved a synergistic suite of hydrolytic enzyme systems to adapt to long-term environmental pressures [42]. Therefore, this study employed directional enrichment to isolate three strains from soil subjected to long-term crop residue amendment. These strains exhibit lignocellulosic degradation capabilities at low temperatures: *Stenotrophomonas* sp. (X24), *Flavobacterium* sp. (X26), and *Erwiniaceae bacterium* (X27). All three strains exhibited remarkable lignocellulosic degradation capabilities at low temperatures.

These findings are consistent with previous reports, which demonstrate that bacterial strains belonging to these genera are known to possess cellulolytic enzyme systems and the ability to degrade biomass. For instance, *S. maltophilia* RSI6 [43] and *F. ginsengisoli* S09 [44] have been reported to produce a variety of lignocellulolytic enzymes. Under moderate temperature conditions (30 °C), *Flavobacterium* sp. Y13 achieves a lignin degradation rate of 61.7% [45]. However, this study provides distinct experimental evidence confirming that *Flavobacterium* sp. (strain X26) and the unclassified *Erwiniaceae* strain X27 exhibit efficient lignocellulose degradation capabilities under low-temperature conditions. Notably, strain X27 exhibited the highest capacity for cellulolytic enzyme production. These findings broaden the known diversity of *Flavobacterium* species and introduce a potentially novel taxonomic lineage within the family *Erwiniaceae* capable of low-temperature lignocellulose degradation. This trait may be attributed to the regulation of cell membrane fluidity, the expression of cold shock proteins, and the accumulation of intracellular antifreeze substances [46], thereby ensuring the normal operation of basal metabolism and enzymatic systems under low-temperature conditions [47]. However, despite the notable performance of strain X27, its individual contribution to straw degradation remains limited, indicating that reliance on a single microbial strain is insufficient to achieve efficient breakdown of complex substrates at low temperatures.

In recent years, an increasing body of research has demonstrated that co-culture systems involving multiple microbial strains often outperform monocultures in various biocatalytic processes [48]. Therefore, this study systematically assembled the three bacterial strains to generate four synthetic microbial mixtures (A, B, C, and D). The results indicate that the three-strain mixture (Mixture C), assembled in equal proportions, exhibits the most effective lignocellulosic biomass degradation capacity, suggesting a high degree of synergistic cooperation among the strains [49]. Specifically, utilizing a 5% inoculum size of Mixture C (pH 7.0) incubated at 12 °C for 45 days, a degradation efficiency of 41.11 ± 1.90% was achieved in the liquid fermentation of corn straw. This significant improvement suggests a form of biological cooperation [50] that may involve metabolic cross-feeding and functional complementarity [51].

The fact that the three strains in the synthetic microbial mixture belong to different genera, suggesting that they may collaboratively degrade lignocellulosic biomass in straw through the secretion of complementary enzymatic systems [52]. The assessment of initial degradation capacity of amorphous cellulose via CMCase activity indicated no significant difference between the three-strain mixture and Mixture D (X26 + X27), suggesting that the *Flavobacterium* sp. and unclassified *Erwiniaceae* strains are the key producers of CMCase [53]. However, when evaluating the comprehensive degradation performance of native crystalline cellulose (characterized by FPase) and the key step of alleviating product inhibition (β-glucosidase activity) [54], the three-strain mixture exhibited significantly higher activity than all other experimental groups. This finding suggests that strain *Stenotrophomonas* sp. functions as an essential “synergist” [55]. Although *Stenotrophomonas* sp. may not be the primary producer of CMCase, its presence is crucial for achieving complete saccharification of straw substrates [56]. Therefore, the outstanding performance of the three-strain mixture essentially reflects the successful emulation of a microecosystem. By Assembling a complete and efficient degradation metabolic pathway [57], this approach overcomes the metabolic bottlenecks typically encountered by individual microorganisms when degrading complex substrates, thereby demonstrating overall robustness and high efficiency [58]. However, in multi-species systems, variations in growth rates inevitably lead to dynamic shifts in community structure during fermentation. Therefore, to fully maximize the catalytic potential of the consortium, future investigations should focus on optimizing inoculation ratios to better align with the differential growth rates and metabolic requirements of *Stenotrophomonas* sp. X24, *Flavobacterium* sp. X26, and the unclassified *Erwiniaceae* strain X27.

The results from the single-factor optimization experiments of the three-strain mixture indicated that its CMCase activity increased with rising temperature up to 12 °C, where it reached a peak before rapidly declining within the tested range of 4–20 °C. This behavior exemplifies the mixture’s characteristic psychrotolerance [59], further confirming that the three bacterial strains within the mixture and their cellulase systems have evolved extensive adaptation to low-temperature environments [60]. Similarly, the optimal pH of 6.0 indicates that the three-strain mixture exhibits the highest metabolic activity in a mildly acidic environment. This finding aligns with the typical phenomenon observed during lignocellulose degradation, where the accumulation of organic acids causes a local decrease in pH [61]. Furthermore, analysis of the kinetic curves relating inoculum size and incubation time revealed that a 4% inoculum size serves as a critical threshold for balance. This level ensures an initial biomass sufficient to reduce lag phase duration [62], while preventing excessive inoculum densities (>4%) that could lead to premature nutrient depletion and rapid accumulation of inhibitory metabolites [63]. In summary, this study systematically elucidates the kinetic characteristics and environmental response patterns of the microbial mixture, successfully establishing an efficient, stable, and controllable lignocellulosic biomass saccharification bioprocess.

Despite the promising degradation efficiency demonstrated by the three-strain mixture, several limitations of this study should be acknowledged. First, the mixture was prepared using a fixed 1:1:1 volumetric ratio. This simplified inoculation strategy did not account for the varying growth rates of the three strains. Consequently, the current ratio may not be optimal for maximizing synergistic cooperation. Future studies should employ statistical optimization methods, such as Response Surface Methodology (RSM), to determine the ideal inoculation ratios for enhanced lignocellulose breakdown. Second, this study was conducted under strictly controlled laboratory conditions using sterile substrates. In natural environments, the degradation process is subject to fluctuating abiotic factors, such as temperature, pH, and moisture, as well as competition from indigenous microbiota. Therefore, further field trials are necessary to validate the stability and ecological adaptability of this bacterial mixture in practical applications.

## 5. Conclusions

We employed a bottom-up approach to assemble a synthetic microbial mixture designed for efficient lignocellulose degradation in cold environments. Specifically, *Stenotrophomonas* sp. X24, *Flavobacterium* sp. X26, and *Erwiniaceae bacterium* X27 were combined in equal proportions to form the synthetic three-strain mixture. Following fermentation at 12 °C for 45 days, the three-strain mixture achieved a net lignocellulosic biomass degradation efficiency of 30.93 ± 1.05% under solid-state fermentation conditions. Under liquid-state fermentation conditions, the net degradation efficiency increased to 41.11 ± 1.90%. Additionally, single-factor optimization experiments identified the optimal parameters for enzyme production: a cultivation period of 72 h, inoculum size of 4%, temperature of 12 °C, and pH of 6.0. Under these conditions, the CMCase activity of the three-strain mixture peaked at 24.51 ± 0.97 U/mL.

## Figures and Tables

**Table 1:** Physiological and biochemical characteristics of the three cold-adapted cellulolytic bacterial strains.

Characteristic	X24	X26	X27
Gram stain	–	–	–
VP test	–	+	–
OF test	+	+	+
Indole test	–	–	–
Gelatin liquefaction test	+	–	+
Catalase test	+	–	+
Oxidase test	–	+	–
Nitrate reduction test	–	+	+
Citrate utilization	+	–	–
Methyl red test	–	+	+
Starch hydrolysis test	–	+	+
Motility	+	–	+
Lipase test	–	+	+

Note: “+” indicates a positive reaction; “−” indicates a negative reaction. Strains were identified as *Stenotrophomonas* sp. (X24), *Flavobacterium* sp. (X26), and *Erwiniaceae bacterium* (X27).

## Data Availability

The original contributions presented in this study are included in the article/Supplementary Material. Further inquiries can be directed to the corresponding authors.

## Supplementary Materials

The following supporting information can be downloaded at: https://www.mdpi.com/article/10.3390/life16030402/s1, Figure S1. Growth curves of the three bacterial strains (*Stenotrophomonas* sp. X24, *Flavobacterium* sp. X26, and *Erwiniaceae bacterium* X27) in CMC liquid medium over 24 h. The cultures were incubated at 30 °C with continuous shaking at 180 rpm. Bacterial growth was monitored by measuring the optical density at 600 nm (A_600_). Data represent the mean ± standard deviation of three independent replicates (*n* = 3). Figure S2. Glucose standard curve generated using the dinitrosalicylic acid (DNS) method. The graph demonstrates the linear relationship between absorbance at 540 nm and glucose concentration (mM) (R^2^ = 0.9901). Data points represent the mean of three independent replicates (*n* = 3). Table S1. Physicochemical properties of the topsoil (0–20 cm) collected from the maize straw returned experimental site. Table S2. Evaluation of low-temperature growth capability for the ten selected cellulose-degrading strains. Table S3. Sequence similarity of the 16S rDNA gene from strain X24 (GenBank accession no. PV793412.1) with corresponding sequences deposited in GenBank. Table S4. Sequence similarity of the 16S rDNA gene from strain X26 (GenBank accession no. PV793411.1) with corresponding sequences deposited in GenBank. Table S5. Sequence similarity of the 16S rDNA gene from strain X27 (GenBank accession no. PV793410.1) with corresponding sequences deposited in GenBank. Table S6. CMCase, FPase, and β-glucosidase activity of different single strains and synthetic mixtures. Table S7. Comparison of experimental parameters between liquid and solid-state fermentation for corn straw degradation.
