# Supplementary material for: Enhanced Low-Temperature Corn Straw Degradation Using a Synthetic Microbial Mixture
Source: Life (Basel). 2026 Mar 2;16(3):402. doi: 10.3390/life16030402 (PMC13027722; doi:10.3390/life16030402)
Supplement: Supplementary file 1 [file life-16-00402-s001.zip › life-4163939-supplementary.pdf]

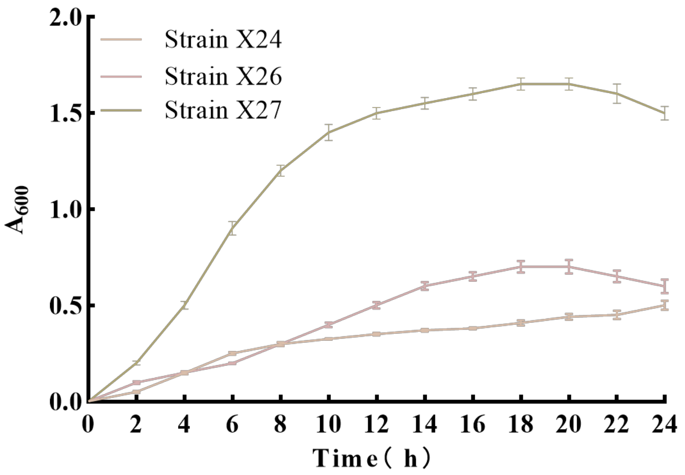

Figure S1. Growth curves of the three bacterial strains (*Stenotrophomonas* sp. X24, *Flavobacterium* sp. X26, and *Erwiniaceae* bacterium X27) in CMC liquid medium over 24 h. The cultures were incubated at 30 °C with continuous shaking at 180 rpm. Bacterial growth was monitored by measuring the optical density at 600 nm (A_{600}). Data represent the mean ± standard deviation of three independent replicates (n = 3).

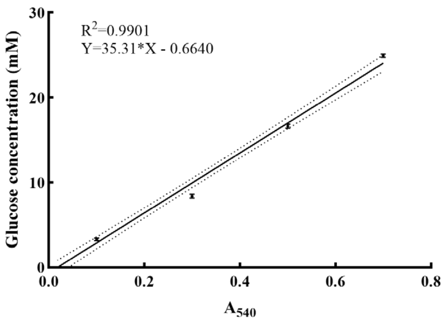

Figure S2. Glucose standard curve generated using the dinitrosalicylic acid (DNS) method. The graph demonstrates the linear relationship between absorbance at 540 nm and glucose concentration (mM) (R^2= 0.9901). Data points represent the mean of three independent replicates (n = 3).

Table S1. Physicochemical properties of the topsoil (0–20 cm) collected from the maize straw returned experimental site

Soil Depth	pH	Moisture Content (%)	Bulk Density (g/cm³)	Total Phosphorus (g/kg)	Total Potassium (g/kg)	Available Phosphorus (mg/kg)	Available Potassium (mg/kg)	Alkali-hydrolyzable Nitrogen (mg/kg)
Topsoil (0-20 cm)	6.04±0.53	16.65±0.01	1.29±0.12	0.64±0.28	26.52±0.92	42.93±6.84	118.74±11.41	96.13±10.34

Note: Data represent the mean ± standard deviation (n = 3). Samples were collected in November from the research station at Shenyang Agricultural University.

Table S2. Evaluation of low-temperature growth capability for the ten selected cellulose-degrading strains.

Strain Number	Temperature（℃）				
	20	16	12	8	4
X4	+	+	+	+	–
X8	+	+	+	–	–
X14	+	–	–	–	–
X17	+	–	–	–	–
X23	+	+	+	+	–
X24	+	+	+	+	+
X26	+	+	+	+	+
X27	+	+	+	+	+
X33	+	–	–	–	–
X43	+	+	+	–	–

Note: These ten strains were selected by ranking their halo-to-colony diameter ratios (D/d) in descending order during the initial carboxymethyl cellulase activity screening. The strains were inoculated onto CMC agar medium. Observation periods were set according to the specific temperatures: 30 days at 4–8 °C, 14 days at 12–16 °C, and 7 days at 20 °C. Growth was determined by the presence of visible colonies as observed by the naked eye. '+' indicates the formation of visible colonies; '–' indicates no visible growth.

Table S3. Sequence similarity of the 16S rDNA gene from strain X24 (GenBank accession no. PV793412.1) with corresponding sequences deposited in GenBank.

Login Number	Type	Homology
CP001111.1	*Stenotrophomonas maltophilia* R551-3, complete genome	99%
AY081992.1	Uncultured bacterium clone KRA30-21 16S ribosomal RNA gene, partial sequence	99%
AJ002814.1	*Stenotrophomonas* sp. 16S rRNA gene, isolate S3	99%
LC136883.1	*Stenotrophomonas* sp. LY-2 gene for 16S ribosomal RNA, partial sequence	99%
KX018308.2	*Stenotrophomonas maltophilia* strain SBR01 16S ribosomal RNA gene, partial sequence	99%
KC581677.1	*Stenotrophomonas maltophilia* strain IARI-ABL-34 16S ribosomal RNA gene, partial sequence	99%
GQ157164.1	Uncultured bacterium clone 16slp101-2c06.p1k 16S ribosomal RNA gene, partial sequence	99%
GQ157162.1	Uncultured bacterium clone 16slp101-3h04.p1k 16S ribosomal RNA gene, partial sequence	99%

Note: The sequence showed high homology (99%) with *Stenotrophomonas* species. Based on this alignment and phylogenetic analysis, strain X24 was identified as *Stenotrophomonas* sp. The sequence length is 1450 bp.

Table S4. Sequence similarity of the 16S rDNA gene from strain X26 (GenBank accession no. PV793411.1) with corresponding sequences deposited in GenBank.

Login Number	Type	Homology
AB681010.1	*Flavobacterium johnsoniae* gene for 16S rRNA, partial sequence, strain: NBRC 15970	99%
CP000685.1	*Flavobacterium johnsoniae* UW101, complete genome	99%
AB078043.1	*Flavobacterium johnsoniae* gene for 16S rRNA, strain:IFO 15970	99%
NR074455.1	*Flavobacterium johnsoniae* strain UW101 16S ribosomal RNA, partial sequence	99%
NR113704.1	*Flavobacterium johnsoniae* strain NBRC 14942 16S ribosomal RNA gene, partial sequence	99%
MG456791.1	*Xanthobacter autotrophicus* strain IMCC34578 16S ribosomal RNA gene, partial sequence	99%
HM224403.1	*Flavobacterium johnsoniae* strain YB7 16S ribosomal RNA gene, partial sequence	99%
NR042496.1	*Flavobacterium johnsoniae* strain UW101 16S ribosomal RNA gene, partial sequence	99%

Note: The sequence showed high homology (99%) with *Flavobacterium* species. Based on this alignment and phylogenetic analysis, strain X26 was identified as *Flavobacterium* sp. The sequence length is 1417bp.

Table S5. Sequence similarity of the 16S rDNA gene from strain X27 (GenBank accession no. PV793410.1) with corresponding sequences deposited in GenBank.

Login Number	Type	Homology
MT733958.1	*Pantoea rodasii* strain JZY4-8 16S ribosomal RNA gene, partial sequence	96%
JX113243.1	*Pantoea rodasii* strain Y36 16S ribosomal RNA gene, partial sequence	96%
MN036533.1	*Pantoea rodasii* strain YZCB2-1 16S ribosomal RNA gene, partial sequence	96%
PP693461.1	*Pantoea rodasii* strain PZ6 16S ribosomal RNA gene, partial sequence	96%
MN036528.1	*Pantoea rodasii* strain HSMGLIZD 16S ribosomal RNA gene, partial sequence	96%
ON597449.1	*Pantoea rodasii* strain iPSB33312 16S ribosomal RNA gene, partial sequence	96%
PQ039734.1	*Pantoea rodasii* strain A8 16S ribosomal RNA gene, partial sequence	96%
ON778745.1	*Pantoea* sp. strain B/B18 16S ribosomal RNA gene, partial sequence	96%

Note: The BLAST search showed approximately 96% homology with *Pantoea* species. Based on this alignment and phylogenetic analysis, strain X27 forms a distinct basal lineage and was identified as an unclassified Erwiniaceae bacterium. The sequence length is 1443 bp.

Table S6. CMCase, FPase, and β-glucosidase activity of different single strains and synthetic mixtures.

	X24	X26	X27	Mixture A	Mixture B	Mixture C	Mixture D
FPase	11.77±0.47d	10.03±0.39e	13.81±0.19c	16.11±0.58b	16.73±0.70b	19.17±1.40a	16.34±0.79b
CMCase	17.23±0.85d	13.43±0.55e	20.85±0.87c	22.25±1.09bc	22.99±0.57ab	23.91±0.57a	22.74±1.01ab
β-glucosidase	8.24±0.23d	6.14±0.23e	9.14±0.30c	9.25±0.31c	10.29±0.29b	12.80±0.62a	10.67±0.47b

Note: Mixtures A–D represent mixture of strains as follows: A (X24 + X26), B (X24 + X27), C (X24 + X26 + X27), and D (X26 + X27). The strains are defined as: X24 (*Stenotrophomonas* sp.), X26 (*Flavobacterium* sp.), X27 *(Erwiniaceae* bacterium). Values represent mean ± SD (n=3). Different letters indicate significant differences ($p < 0.05$, ANOVA with Tukey's test).

Table S7. Comparison of experimental parameters between liquid and solid-state fermentation for corn straw degradation.

Parameter	Liquid Fermentation	Solid-State Fermentation
Straw state	40-mesh ground straw	2–3 cm straw segments
Substrate Mass (dry weight)	2.0 g	10.0 g
Medium Volume	100 mL	20 mL
Medium Concentration	1× base concentration	5× enriched concentration
Inoculum Volume	5.0 mL	2.0 mL
Temperature / Duration	12 °C / 45 days	12 °C / 45 days
